# Tumor Secretion of CCL22 Activates Intratumoral Treg Infiltration and Is Independent Prognostic Predictor of Breast Cancer

**DOI:** 10.1371/journal.pone.0076379

**Published:** 2013-10-04

**Authors:** Ya-Qing Li, Fang-Fang Liu, Xin-Min Zhang, Xiao-Jing Guo, Mei-Jing Ren, Li Fu

**Affiliations:** 1 Department of Breast Cancer Pathology and Research Laboratory, Tianjin Medical University Cancer Institute and Hospital, National Clinical Research Center of Cancer, Tianjin, China; 2 Key Laboratory of Breast Cancer Prevention and Therapy, Tianjin Medical University, Ministry of Education, Tianjin, China; 3 Key Laboratory of Cancer Prevention and Therapy, Tianjin, China; 4 Department of Pathology and Laboratory Medicine, Temple University Hospital, Philadelphia, Pennsylvania, United States of America; 5 2011 Collaborative Innovation Center of Tianjin for Medical Epigenetics, Tianjin, China; Sapporo Medical University, Japan

## Abstract

It has been reported that dense intratumoral infiltration of Foxp3 ^+^Tregs (Tregs) was an independent factor for poor prognosis of breast cancer (BC) patients. However, the cytokines activating the Treg infiltration are not known. This study was undertaken to evaluate the role of CCL22 and TGF-β1 in this cascade and their prognostic significance for BC patients. 417 cases of invasive breast cancer were selected from the prior study cohort and the expressions of CCL22 and TGF-β1 were assessed by immunohistochemistry. It was identified that tumor secretion of CCL22 was positively correlated with the intratumoral Treg infiltration (*P*<0.0001), but its association with lymphoid aggregates surrounding the tumor was not proven to be significant (P=0.056). Moreover, CCL22 expression was found to be associated with the tumor histological features known to be related with unfavorable prognosis of patients, including high histological grade (*P*<0.0001), negative ER (*P*<0.0001), negative PR (*P*=0.001), and HER2 amplification (*P*=0.028). Similar to intratumoral Treg infiltrates, CCL22 tumor secretion correlated with the prognosis of the molecular subtypes of breast carcinoma (*P*<0.0001). Univariate analysis revealed CCL22 to be an independent prognostic factor for overall survival (OS, *P*<0.0001) and progression-free survival (PFS, *P*<0.0001) of BC patients that were confirmed by multivariate analysis (P=0.011 and P=0.010 respectively). In contrast, although TGF-β1 expression was positively correlated with both Tregs infiltrates into the tumor bed and lymphoid aggregates surrounding the tumor (*P*=0.023; *P*=0.046, respectively), its expression was not significantly associated with the molecular subtypes of breast carcinoma and the prognosis of the patients. Our study indicates that both CCL22 and TGF-β1 are candidate chemoattractants for intratumoral Foxp3 ^+^Tregs infiltration; however, unlike the later, CCL22 is an independent prognostic predictor of BC patients, and it therefore may have the potential to serve as a target for immunotherapeutic strategy of BC.

## Introduction

CD4^+^CD25^+^Foxp3^+^regulatory T cells (Tregs) are thought to be the main obstacle tempering antitumor immunity and immunotherapy [1,2]. Their localization and the infiltrating patterns vary in breast cancer (BC) and have different impacts on tumor progression [3,4], and our prior study indicated that intratumoral Treg infiltration was an independent adverse prognostic factor for BC [5]. Tregs are frequently found to accumulate within the tumor mass via recruitment by chemokines; however which cytokines chemotactically activate the Tregs to migrate into the tumor bed and whether these participating cytokines have their own independent role in breast cancer progression remain unknown.

CCL22, also known as macrophage-derived chemokine (MDC), was originally found to be secreted by macrophages and dendritic cells (DC) [6]. Researchers have recently identified that the interaction between natural killer (NK) cells and tumor cells can drive tumor cells to produce CCL22 [7]. CCL22 is one of the ligands for chemokine receptor CCR4 [8-10] that preferentially express on Tregs and is required for intratumoral Treg migration. Within the tumor bed, Tregs appear to expand and become activated by tumor-associated antigens as well as normal self-antigens expressed by tumor cells, and result in inhibition of activated T cells. A few studies have indicated that CCL22 secreted by solid tumor cells is responsible for accumulation of Foxp3 ^+^Tregs in ovarian [6], prostate [11], gastric [12], esophageal [13], and breast carcinomas [7,9]. This cascade might lead to suppression of the local immune response and to favor tumor survival and growth.

Other studies have revealed that the cytokine transforming growth factor-β1 (TGF-β1) play a vital role in inducing the differentiation of Tregs [14,15] and in mediating suppression of the activation, differentiation and proliferation of immune cells [16,17]. It is secreted by many types of cells, including Tregs and tumor cells. TGF-β1 is a multifunctional cytokine and has several impacts on BC [18]. Ohara et al. [19] have found that the expression of CCL22 and TGF-β1 was upregulated at mRNA level in invasive breast carcinoma and is positively correlated with Foxp3 expression. The study of Gupta et al. [4] found that the levels of intratumoral Treg infiltration were associated with the levels of TGF-β1 expression in BC. However, it is unclear that the biological meaning of TGF-β in recruiting Tregs into the tumor microenvironment. Whether TGF-β1 has independent impacts on prognosis of BC patients also remains to be explored.

The present study was designed to assess the association of CCL22 and TGF-β1 expression in tumor cells with the status of Treg infiltration in invasive breast carcinoma, and in addition, to evaluate their prognostic significance for BC patients.

## Materials and Methods

### Ethics statement

All human breast tissues were collected with written consent from patients prior to participation in the study. The protocols for collection and analysis of the samples were approved by the Institutional Review Board of the Tianjin Medical University Cancer Institute and Hospital, in accordance with the current revision of the Helsinki Declaration.

### Specimen selection and clinical information

417 contiguous cases of invasive breast cancer diagnosed from January to October of 2003 were selected from our prior study cohort [5]. The cases included 337 invasive ductal carcinoma, not otherwise specified type (IDC-NOS), 44 invasive lobular carcinomas (ILC) and 36 cases other histological types. Histological grading was carried out using the modified Bloom and Richardson grading system [20]. All patients were females with an age range from 38 to 92 years (median 52 years). No radiation and/or chemotherapy were offered to any of the patients before surgery. Postoperatively, 378 (90.6%) patients received adjuvant chemotherapy, 187 (44.8%) endocrine therapy, and 161 (38.6%) radiation therapy. The patients were followed up for 5 to 118 months with a median of 76 months, during which 19 (4.6%) patients suffered local or regional tumor recurrence, 76 (18.2%) patients developed distant metastasis, and 34 (8.2%) patients died of tumor.

### Immunohistochemistry for CCL22 and TGF-β1

Formalin-fixed, paraffin-embedded serial tissue sections from each case were obtained. Immunohistochemistry (IHC) for CCL22 and TGF-β1 were performed using standard procedures. In brief, 5 cm tissue sections were subsequently dewaxed and rehydrated using xylene and graded alcohol washes. Antigen retrieval was performed at 121°C for 2 min, using citrate buffer, pH 6.0. After serial blocking with hydrogen peroxide and normal goat serum, the sections were incubated with primary monoclonal antibody against CCL22 (Abcam, ab9847, 1:40 dilution), TGF-β1 (Newmark, ab0238, 1:40 dilution) for 16 h at 4°C. The sections then were sequentially incubated with biotinylated goat anti-mouse immunoglobulin and peroxidase-conjugated strep-tavidin (DAKO). The enzyme substrate was 3-3’-diaminobenzidine tetra-hydrochloride. Incubation of sections with phosphate-buffered saline only served as negative controls.

### Assessment of CCL22 and TGF-β1 Cytokines

Both CCL22 and TGF-β1 IHC demonstrated a cytoplasmic staining of the tumor cells with varying intensity, and only occasional staining of other types of cells are noted. Cases were scored negative if no staining of the cytoplasm of tumor cells was found (0 = negative), and positive if cytoplasmic staining of tumor cells was presented. The positive cases showed an universal staining in all the tumor cells and were subdivided into two groups based on the intensity of staining (1=weak; 2=strong).

### Assessment of Tregs

Tregs infiltrates into the tumor bed and lymphoid aggregates surrounding the tumor were re-analyzed using the previously slides for Foxp3, accordance to prior set of criteria [5]. Cutoff on the cellular density of Tregs for dividing the subgroups was the median value (median 11 vs. 36). Further, all cases were divided into three groups by two factors: cases without CCL22 expression and with low density of Foxp3 ^+^Tregs in the tumor bed were defined as CCL22^-^Foxp3^low+^ group, cases with CCL22 expression and with high density of Foxp3 ^+^Tregs in the tumor bed were defined as CCL22^+^Foxp3^high+^ group, and the remaining cases as CCL22^-^Foxp3^high+^/CCL22^+^Foxp3^low+^ group.

### Immunohistochemistry for Molecular Subtypes

Previously tissue sections stained for ER, PR, HER2, CK5/6, EGFR and Ki-67 of the selected cases were re-analyzed using the prior set of criteria [5]. For ER, PR, and HER2, the immunohistochemistry was scored according to the ASCO/CAP Guideline [21]. Cases with a HER2 score of 2+ were further evaluated by fluorescence in situ hybridization (FISH) using Vysis kit (Vysis, Inc., Downers Grove, IL). Interpretation and scoring of Ki-67, EGFR, and CK5/6 staining described by Cheang et al [22] were adopted in our study, and BC molecular subtypes were categorized as follows: luminal A (ER positive and/or PR positive, and Ki-67<14%), luminal B (ER positive and/or PR positive and Ki-67≥14%), luminal-HER2 (ER positive and/or PR positive and HER2 positive), HER2 enriched (ER negative, PR negative and HER2 positive), and basal-like (ER negative, PR negative, HER2 negative, and EFGR and/or CK5/6 positive). In addition, triple-negative tumors (TNP, negative for ER, PR and HER2) that were negative for both EGFR and CK5/6 were classified as TNP-nonbasal [23].

### Statistic analysis

The SPSS 15.0 software package was used for statistical analyses. Mann–Whitney U test, χ^2^ test were performed for group comparisons and continuous data were compared using analysis two-tailed t-tests. Cumulative survival (progression-free survival, PFS; overall survival, OS) time was calculated by the Kaplan-Meier method and analyzed by the log-rank test. Univariate and multivariate analyses were based on the Cox proportional hazards regression model. Correlations were studied with Spearmen’s correlation. A 2-sided P < 0.05 was considered statistically signiﬁcant in the analyses.

## Result

### The characteristics of BC patients and Treg infiltration of the tumor tissue

The characteristics of BC patients were summarized in Table 1. 130 (31.2%) patients presented with higher grade tumors. All patients underwent surgical excision with axillary lymph node dissection. 236 (56.6%) patients had lymph node metastasis, 193 (46.3%) of the tumors were positive for ER, 178 (42.7%) were positive for PR, and 154 (36.9%) showed HER2 overexpression. The molecular subtypes of the tumors revealed that the luminal A subtype (25.2%) was most prevalent, followed by basal-like (23.7%), luminal HER2 (18.5%), and HER2- enriched (18.5%) and luminal B subtype (14.1%). No TNP-nonbasal tumors were observed in this study due to its rarity.

**Table 1:** Clinicopathologic characteristics of BC patients.

		**Total cases**	
**Characteristics**		**n**	**%**
No. of patients		417	100
Age (years)	<50	177	42.4
	≥50	240	57.6
Tumor size (cm)	≤2	103	24.7
	>2	314	75.3
Lymph node	Negative	181	43.4
	Positive	236	56.6
Histological grade	i	27	6.5
	ii	260	62.4
	iii	130	31.2
ER status	Negative	224	53.7
	Positive	193	46.3
PR status	Negative	239	57.3
	Positive	178	42.7
HER2 status	Negative	263	63.1
	Positive	154	36.9
Molecular subtypes	Luminal A	105	25.2
	Luminal B	59	14.1
	Luminal HER2	77	18.5
	HER2-enriched	77	18.5
	Basal-like	99	23.7

High density of Tregs infiltration within the tumor bed was found to be an unfavorable prognostic factor measured by OS (*P*<0.0001) and PFS (*P*<0.0001), but not in lymphoid organs. Significant differences in the density of Treg infiltration among the molecular subtypes were also revealed (*P*<0.0001) (Table 2). These results were consistent with our previous analysis [5].

**Table 2:** Expression of CCL22,TGF-β1 and Foxp3^+^Tregs among BC molecular subtypes.

	**Luminal A**	**Luminal B**	**Luminal HER2**	**HER2-enriched**	**Basal-like**	P*
FOXP3^+^ (Median^a^)	4	8	10	10	22	<0.0001
CCL22^+^	51.4	62.7	76.7	80.6	85.9	<0.0001
(No. of patients %)						
TGF-β1^+^	65.7	72.9	59.8	71.5	69.7	0.198
(No. of patients %)						

a Cell count per field (cells/0.0625 mm^2^)

* *P* values were calculated by Spearman’s Rank-Correlation test (n = 417)

### Tumor expression of CCL22 and TGF-β1

**Figure 1:**
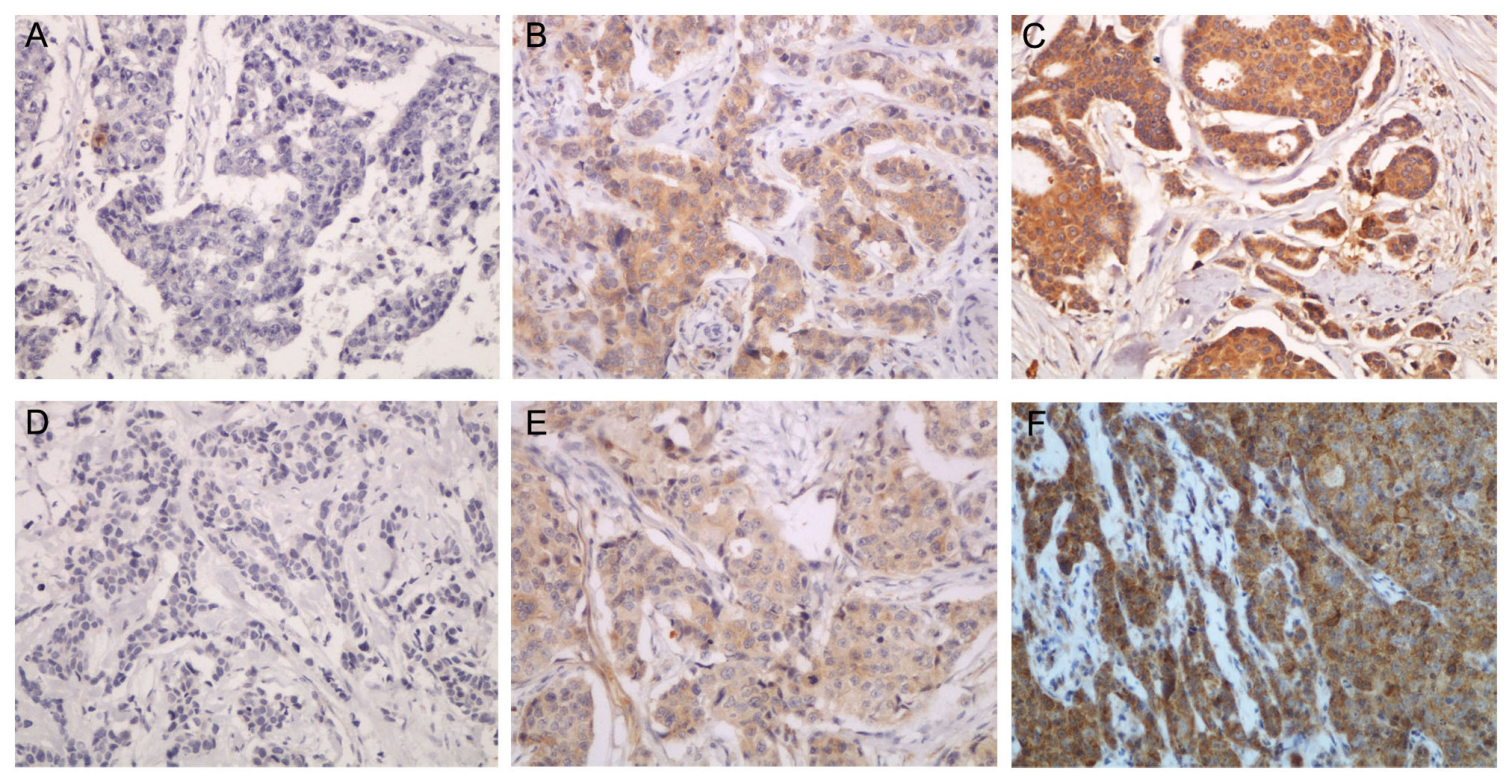
CCL22 and TGF-β1 expression in BC. (A) Negative expression of TGF-β1 in BC (×200). (B) Weak expression of TGF-β1 in BC (×200). (C) Strong expression of TGF-β1 in BC (×200). (D) Negative expression of CCL22 in BC (×200). (E) Weak expression of CCL22 in BC (×200). (F) Strong expression of CCL22 in BC (×200).

297 (71.2%) and 282 (67.6%) of breast carcinomas showed positive immunohistochemical staining for CCL22 and TGF-β1 respectively (Figure 1). The upregulated CCL22 expression correlated with the prognostically unfavorable features of tumors, including high histological grade (*r*_s_=0.210, *P*<0.0001), negative ER (*r*_s_=-0.212, *P*<0.0001), negative PR (*r*_s_=-0.167, *P*=0.001), and HER2 overexpression (*r*_s_=0.108, *P*=0.028). No signiﬁcant associations were identified between the expression of CCL22 and patient age, tumor size, or lymph node status. In contrast, no significant association was identified between TGF-β1 expression and pathologic features of the tumors (Table 3).

**Table 3:** CCL22 or TGF-β1 expression and pathological features of BC.

**Characteristic**	**CCL22**						**TGF-β1**				
	**Negative**	**Low**	**High**	**r_s_**	P*		**Negative**	**Low**	**High**	**r_s_**	P*
No. of patients	120 (28.8)	166 (39.8)	131 (31.4)				135 (32.4)	152 (36.5)	130 (31.2)		
Age (years)				0.016	0.740					-0.037	0.448
< 50	54 (30.5)	67 (37.9)	56 (31.6)				54 (30.5)	65 (36.7)	58 (32.8)		
≥50	66 (27.5)	99 (41.3)	75 (31.3)				81 (33.8)	87 (36.3)	72 (30.0)		
Tumor size (cm)				0.048	0.327					0.034	0.530
≤2	33 (32.0)	41 (39.8)	29 (28.2)				36 (35.0)	37 (35.9)	30 (29.1)		
> 2	87 (27.7)	125 (39.8)	102 (32.5)				99 (31.5)	115 (36.6)	100 (31.8)		
Lymph node				-0.038	0.435					0.033	0.498
Negative	47 (26.0)	76 (42.0)	58 (32.0)				64 (35.4)	61 (33.7)	56 (30.9)		
Positive	73 (30.9)	90 (38.1)	73 (30.9)				71 (30.1)	91 (38.6)	74 (31.4)		
Histological grade				0.210	<0.0001					0.077	0.114
i	13 (48.1)	10 (37.0)	4 (14.8)				7 (25.9)	12 (44.4)	8 (29.6)		
ii	82 (31.5)	108 (41.5)	70 (26.9)				92 (35.4)	95 (36.5)	73 (28.1)		
iii	25 (19.2)	48 (36.9)	57 (43.8)				36 (27.7)	45 (34.6)	49 (37.7)		
ER status				-0.212	<0.0001					-0.070	0.153
Negative	50 (22.3)	84 (37.5)	90 (40.2)				71 (31.7)	73 (32.6)	80 (35.7)		
Positive	70 (36.3)	82 (42.5)	41 (21.2)				64 (33.2)	79 (40.9)	50 (25.9)		
PR status				-0.167	0.001					-0.023	0.636
Negative	58 (24.3)	90 (37.7)	91 (38.1)				78 (32.6)	82 (34.3)	79 (33.1)		
Positive	62 (34.8)	76 (42.7)	40 (22.5)				57 (32.0)	70 (39.3)	51 (28.7)		
HER2 status				0.108	0.028					-0.032	0.512
Negative	87 (33.1)	99 (37.6)	77 (29.3)				82 (31.2)	97 (36.9)	84 (31.9)		
Positive	33 (21.4)	67 (43.5)	54 (35.1)				53 (34.4)	55 (35.7)	46 (29.9)		

* *P* values were calculated by Spearman’s Rank-Correlation test (n = 417)

### Association of CCL22 and TGF-β1 expression with Foxp3 expression

Expression of CCL22 was significantly associated with the infiltration of Tregs in the tumor bed (*r*_s_=0.282, *P*<0.0001), but not with those aggregates surrounding the tumor (*r*_s_=0.094, *P*=0.056). Positive correlation was also observed between expression of TGF-β1 and both Treg infiltrates the tumor bed and lymphoid aggregates surrounding the tumor (*r*_s_=0.111, *P*=0.023; *r*_s_=0.098, *P*=0.046, respectively) (Table 4).

**Table 4:** Correlation of CCL22 or TGF-β1 and Foxp3^+^Tregs infiltration.

	**CCL22 (%)**						**TGF-β1 (%)**				
	**negative**	**weak**	**strong**	**r_s_**	p*		**negative**	**weak**	**strong**	**r_s_**	p*
Foxp3^+^											
Tumor bed				0.282	<0.0001					0.111	0.023
Low<11	84 (39.8)	83 (39.3)	44 (20.9)				80 (37.9)	72 (34.1)	59 (28.0)		
High≥11	36 (17.5)	83 (40.3)	87 (42.2)				55 (26.7)	80 (38.8)	71 (34.5)		
Peritumoral				0.094	0.056					0.098	0.046
Low<36	47 (27.3)	88 (51.2)	37 (21.5)				66 (38.4)	58 (33.7)	48 (27.9)		
High≥36	73 (29.8)	78 (31.8)	94 (38.4)				69 (28.2)	94 (38.4)	82 (33.5)		

* *P* values were calculated by Spearman’s Rank-Correlation test (n = 417)

### CCL22 and TGF-β1 expression and prognosis of BC patients

**Figure 2:**
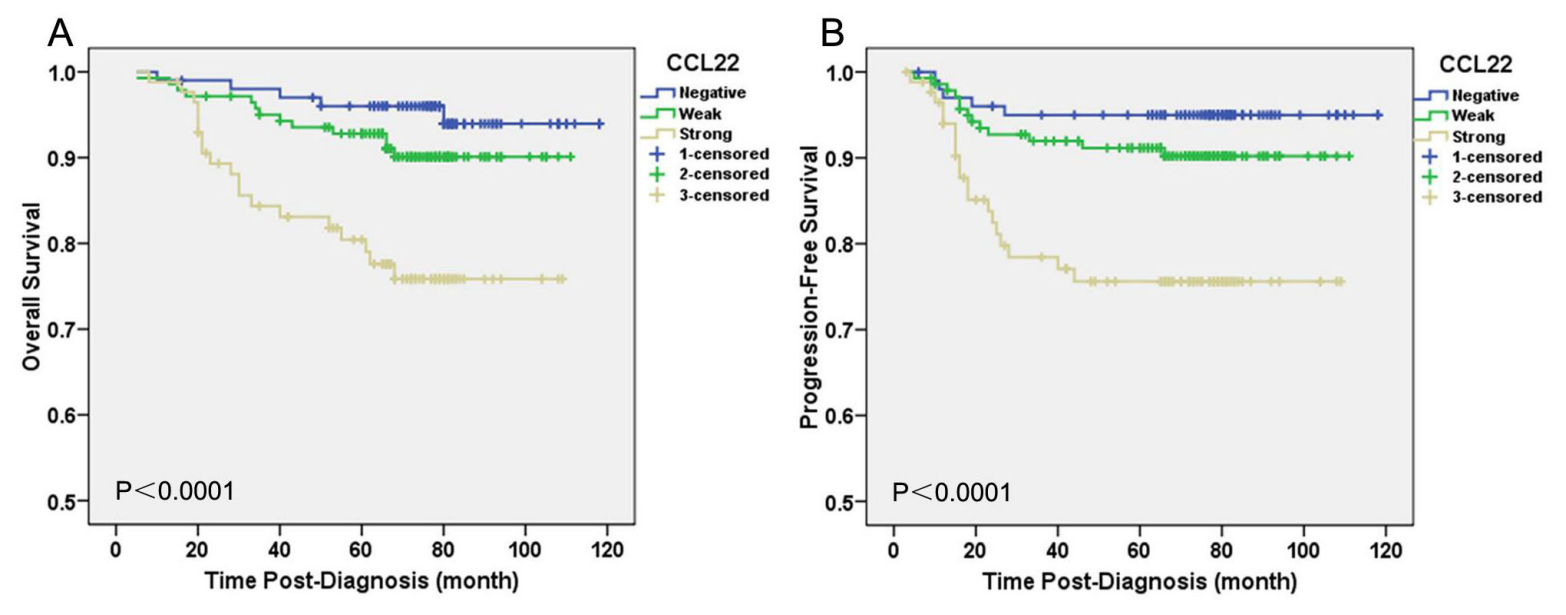
Prognostic significance of CCL22 Expression in BC. Kaplan–Meier curves of overall survival (OS) (A) and progression-free survival (PFS) (B) for CCL22 expression in BC to show the association of CCL22 expression with reduced OS (P<0.0001) and PFS (P<0.0001). P values were calculated by the log-rank test.

**Figure 3:**
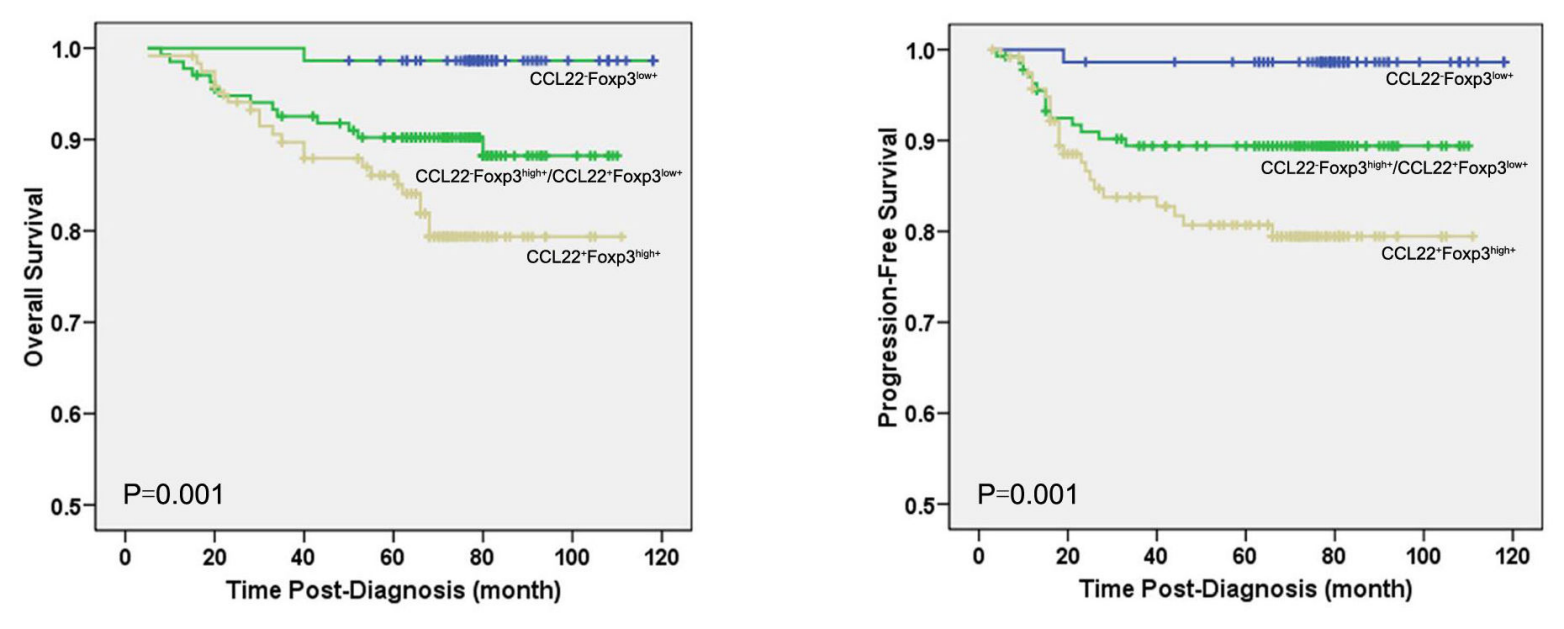
Expression of both CCL22 and Foxp3 ^+^Tregs infiltration in the tumor bed was associated with BC prognosis. Kaplan-Meier curves are shown for overall survival (OS) (A) and progression-free survival (PFS) (B) was stratified by expressions of two factors to divide the patients into three subsets, CCL22^-^Foxp3^low+^ group, CCL22^-^Foxp3^high+^/CCL22^+^Foxp3^low+^ group, and CCL22^+^Foxp3^high+^ group. The CCL22^+^Foxp3^high+^ group was associated with both shorter OS (P=0.001) and PFS (P=0.001) than CCL22^-^Foxp3^high+^/CCL22^+^Foxp3^low+^ group, and CCL22^-^Foxp3^low+^ group.

High expression of CCL22 was found to be an unfavorable prognostic factor measured by OS (*P*<0.0001) and PFS (*P*<0.0001) (Figure 2). Moreover, as the significant indicator identified in univariate analysis, CCL22 was selected to enter into the Cox proportional hazards model for multivariate survival analyses. The results revealed that CCL22 was independent adverse predictor of OS (*P*=0.011) and PFS (*P*=0.010) (Table 5 and Table 6). Further, since there was a significant positive correlation between CCL22 and intratumoral Foxp3^+^Tregs expression, we tried to determine whether the two factors are interacting together to affect the prognosis of BC patients. The patients who were in CCL22^+^Foxp3^high+^ group had a significantly shorter OS (*P*=0.001) and PFS (*P*=0.001) than CCL22^-^Foxp3^high+^/CCL22^+^Foxp3^low+^ group and CCL22^-^Foxp3^low+^ group (Figure 3). In contrast, TGF-β1 expression was not proven to be significantly associated with the prognosis of BC patients.

**Table 5:** Univariate and multivariate analyses (logistic regression) of pathological features and cytokines for OS in BC.

	**Univariate Analysis**				**Multivariate Analysis**		
**Factors**	**OR**	**95% CI**	***P***		**OR**	**95% CI**	***P***
Age (<50 vs. ≥50)	1.364	0.603-3.087	0.456		—	—	—
Tumor size, cm (≤2 vs. >2)	1.765	0.734-4.240	0.204		—	—	—
Node status (cN- vs. cN+)	4.111	1.706-9.904	0.002		4.081	1.692-9.845	0.002
Tumor grade (I vs.II vs. III)	3.157	1.718-5.799	<0.0001		2.326	1.235-4.380	0.009
CCL22 (negative vs. weak vs. strong)	2.435	1.523-3.893	<0.0001		1.892	1.160-3.086	0.011
TGF-β1 (negative vs. weak vs. strong)	1.327	0.873-2.016	0.185		—	—	—

95% CI, 95% confidence interval; OR, odds ratio.

**Table 6:** Univariate and multivariate analyses (logistic regression) of pathological features and cytokines for PFS in BC.

	**Univariate Analysis**				**Multivariate Analysis**		
**Factors**	**OR**	**95% CI**	***P***		**OR**	**95% CI**	***P***
Age (<50 vs. ≥50)	1.408	0.622-3.187	0.411		—	—	—
Tumor size, cm (≤2 vs. >2)	1.795	0.747-4.312	0.191		—	—	—
Node status (cN- vs. cN+)	4.193	1.740-10.102	0.001		4.174	1.730-10.070	0.001
Tumor grade (I vs.II vs. III)	3.103	1.693-5.688	<0.0001		2.267	1.207-4.260	0.011
CCL22 (negative vs. weak vs. strong)	2.429	1.519-3.886	<0.0001		1.904	1.165-3.109	0.010
TGF-β1 (negative vs. weak vs. strong)	1.305	0.858-1.985	0.213		—	—	—

95% CI, 95% confidence interval; OR, odds ratio.

### Correlation between CCL22 and TGF-β1 expression with molecular subtypes of tumor

Significant difference of CCL22 expression was identified among the molecular subtypes (*P*<0.0001). The positivity of CCL22 expression was lowest in luminal A (51.4%), and increased gradually to 62.7% in luminal B, 76.7% in luminal-HER2, 80.6% in HER2-enriched, and to 85.9%, in basal-like subtype, corresponding to the aggressiveness of the biological behavior. The same relationship was not proven in the TGF-β1 expression (*P*=0.198) (Table 2).

## Discussion

Tregs are believed to play an important role in tumor immunity and investigation on the patterns of its infiltration and its clinical impact on patients has become an emerging interest [9,23-25]. However, the mechanisms through which the Tregs are recruited into the tumor microenvironment are not known in BC, particularly in regarding which cytokines are involved in the chemotactic process, and what roles these cytokines actually play. It is reasonable to hypothesize that cytokines secreted by tumor cells are candidate recruiters of Tregs. Several studies have demonstrated that CCL22 can selectively recruit Tregs into the tumor microenvironment, resulting in the accumulation of Tregs [6-12]. So far only a few studies have evaluated the expression of cytokines within breast tumors related with Treg infiltration. The results of Faget J et al. [7] and Gobert et al. [9] have suggested that tumor-derived CCL22 might be an important cytokine in the Treg recruitment of BC. Ohara et al. [19] reported that the levels of CCL22 and TGF-β1 mRNA were elevated in invasive breast cancer tissues, and their expressions positively correlated with the level of Foxp3 mRNA. Gupta et al. [4] found that intratumoral Foxp3 mRNA and protein expression were associated with the intratumoral expression of TGF-β1. Although these studies compared the cytokines in normal, ductal carcinoma in situ (DCIS) and invasive carcinomas, their associations with Treg infiltration at different locations within the tumor microenvironment were not addressed. Our prior study demonstrated that the density of Treg infiltration within the tumor bed was an independent prognostic factor and increased Treg infiltrates was associated with poor prognosis of BC patients, while decreased ratio of cytotoxic T lymphocytes (CTLs) and Tregs aggregates surrounding the tumor was a poor prognostic indicator of patients [5]. Therefore, it becomes part of our interest to assess the expression of these cytokines corresponding to the sub-locations of the Tregs in tumor. Our findings demonstrated that the expressions of both tumor-secreted CCL22 and TGF-β1 were positively correlated with the intratumoral Tregs at the immunohistochemistry level. However, in the tissues surrounding tumor, only the tumor-derived TGF-β1 expression was significantly associated with the Treg infiltration. These results indicated that both tumor-derived CCL22 and TGF-β1 were involved in the chemotaxis of Tregs in the tumor bed, but the role of CCL22 in the recruitment of Tregs into surrounding the tumor was minimal.

Therefore, we speculated Foxp3 ^+^Tregs aggregate surrounding the tumor of BC were not merely dependent on CCL22 but via other chemotaxis. CCR4 is known to be down regulated on Tregs after their sensitization to CCL22 but other chemokine receptors, such as CCR6, which are expressed on these Tregs, which is interaction with CCL20 production by breast tumor cells [26,27]. Studies showed that the majority of Foxp3^+^Tregs accumulating in the tumor microenvironment were expressing inducible costimulator (ICOS). While the expansion and the suppressive function of these Foxp3^+^ICOS^+^Treg cells was strictly dependent on ICOS-ligand (ICOS-L) stimulation provided by tumor plasmacytoid dendritic cells (pDCs) in ovarian cancer [28] and BC [29]. Researchers have also confirmed that tumor-associated pDC strongly correlated with Tregs and that tumor-associated pDC altered functionality (loss of IFN-α secretion) was associated with Foxp3 ^+^Tregs accumulation within BC [30]. Therefore, these mechanisms added to explain Tregs aggregate surrounding the tumor may be via tumor-secreted CCL20 after interaction with DC subsets.

Besides the chemotactic effect of these cytokines, it has also been reported that Tregs can be converted from non-Tregs by high levels of tumor-derived TGF-β in culture [31]. And TGF-β could convert functional DC into dysfunctional ones in tumor microenvironment via either directly or indirectly modulating DC functionality, which in turn stimulate Tregs differentiation and expansion [32]. In addition, Yang et al. [33] studied on TGF-β-miR-34a-CCL22 signaling in hepatocellular carcinoma and revealed that the activity of TGF-β could suppress the expression of miR-34a, resulting in the enhanced production of the chemokine CCL22 and recruitment of Tregs. This model was for the first time from a new point of view to clarify the potential mechanism of TGF-β and Treg’s accumulation. It is possible that similar mechanism exists in BC where Treg’s accumulation may be induced through the interaction of TGF-β1 and CCL22. Therefore, Tregs may mainly via the expression of cytokines and receptors interaction with cytokines in tumor microenvironment for producing indirect role to tumor cells, then affect the progression of BC. It is obvious that the mechanisms through which Treg infiltrating in BC tissue are far more complicated than our current understanding. Further studies are warranted to explore the underlined mechanisms that will hopefully lead to the establishment of a working model for BC.

Another interesting question is whether the significant cytokines within the cascade toward activating Tregs to infiltrate the tumor tissue would impose independent impact on BC patient’s outcome. We therefore analyze the expressions of CCL22 and TGF-β1 in relationship with prognosis of the patients. Similar to Foxp3 expression in BC [5], upregulation of CCL22 expression in tumor cells was significantly more common in carcinomas with unfavorable histological features, such as high tumor grade, ER-negativity, PR-negativity and HER2-overexpression. Univariate analysis showed that upregualted CCL22 expression was significantly associated with reduced OS and PFS, and multivariate analysis confirmed it to be an independent indicator for poor prognosis. In addition, since expression of CCL22 was significantly associated with intratumoral Treg infiltration, we try to assess the joint expression of the two factors are interacting together to affect the prognosis of BC patients. The results showed that CCL22^+^Foxp3^high+^ in the tumor bed was predicted a worse survival, which further confirmed that CCL22 was in interaction with Tregs. However, no significant association was identified between its expression and tumor size and lymph node metastasis. Similar findings were described between Treg infiltrates and these two important prognostic factors of BC patients in our prior study [5]. In contrast, analysis of TGF-β1 failed to identify significant associations with these unfavorable tumor features, and, it was not surprising that its expression was insignificantly associated with patient’s OS and PFS in univariate analysis.

Molecular subtypes of invasive breast cancer has been proven to link to the prognosis of BC patients, and a few studies have focused on the correlation between Tregs and molecular subtypes of BC [34-37]. Our previous study demonstrated that the density of Treg infiltrates within the tumor bed significantly increased as the prognosis of the subtypes (luminal A, luminal B, luminal HER2, HER2-enriched, and basal-like subtype) was ranked from well to poor [5], and recent study was similar to that. However, a 175 cases study by West NR et al. [37] suggested that tumor-infiltrating Foxp3^+^lymphocytes were linked to good clinical outcome in basal-like BC subtype, which seemingly contradicts the results of our study. This may be due to the fewer cases in their study or a geographic predilection. The main reason that caused the disparate results may be the differences of the tissue volume selected. We used full block-face tissue sections instead of tissue array to select the areas with abundant TILs in the tumor bed and lymphoid aggregates surrounding the tumor, thereby avoiding the problem that insufficient number of Tregs in the single location of different BC subtypes.

Interestingly, a similar association was identified at current study between tumor-derived CCL22 expression and the molecular subtypes of BC. Significant differences in the status of CCL22 expression among the five molecular subtypes were revealed, and the expression was increased corresponding to molecular subtypes in the prognostic order from well to poor. In contrast, no significant TGF-β1 expression among the molecular subtypes was identified. Our results strongly argue for that despite the fact that both CCL22 and TGF-β1 involve in the tumor infiltration of Tregs, only CCL22 expression can serve as an independent prognostic predictor of BC patient’s survival. Therefore, immunotherapeutic strategy against CCL22 may reduce the intratumoral Tregs and improve the outcomes of BC, especially for those patients with the tumor subtypes, such as basal-like carcinoma that has only limited therapeutic regimes and will not benefit from endocrine therapy and HER2/neu targeted therapy. However, further study to validate the findings in large scale of samples is required. In addition, exploration on whether the Tregs recruited into the tumor bed by CCL22 and TGF-β1 are heterogeneous populations and may have different roles in tumor immunity would be another interesting study.

In conclusion, the study results indicate that both CCL22 and TGF-β1 are candidate chemoattractants for intratumoral Foxp3 ^+^Tregs infiltration; however, unlike the later, CCL22 is an independent prognostic predictor of BC patients, and it therefore may have the potential to serve as a target for immunotherapeutic strategy for BC patients. Additional studies are required to fully explore the mechanisms involving in the activation of Tregs infiltrates and their accurate roles in BC immunity.
